# Fulminant Hepatitis Due to Enterovirus E25 Systemic Infection in a Pediatric Patient

**DOI:** 10.3390/pathogens15070666

**Published:** 2026-06-25

**Authors:** Silvia Garattini, Lorenza Romani, Luana Coltella, Tommaso Alterio, Stefania Mercadante, Costanza Tripiciano, Maia De Luca, Sara Chiurchiù, Laura Cursi, Francesca Ippolita Calò Carducci, Cristina Russo, Carlo Federico Perno, Alberto Villani, Andrea Pietrobattista, Stefania Bernardi, Laura Lancella

**Affiliations:** 1 Division of Pediatric Infectious Disease, Department for Women’s and Children’s Health, Padua University Hospital, 35128 Padua, Italy; 2 Infectious Disease Unit, Bambino Gesù Children’s Hospital, IRCCS, 00165 Rome, Italy; 3 Microbiology and Diagnostic Immunology Unit, Bambino Gesù Children’s Hospital, IRCCS, 00165 Rome, Italy; 4 Unit of Hepatology and Transplant Clinic, Bambino Gesù Children’s Hospital, IRCCS, 00165 Rome, Italy; 5 Department of Pediatrics, Bambino Gesù Children’s Hospital, IRCCS, University of Rome Tor Vergata, 00165 Rome, Italy

**Keywords:** pediatric acute liver, enterovirus, Epstein–Barr

## Abstract

Pediatric acute liver failure (PALF) is a rare but life-threatening condition characterized by rapid clinical deterioration and high mortality. Viral infections represent a major etiology of PALF, although the causative agent remains unidentified in a substantial proportion of cases. Human Enteroviruses (EVs) are typically associated with self-limiting illnesses; however, they may rarely cause severe systemic disease, including fulminant hepatitis, particularly in neonates and young children. We describe the case of a 4-year-old previously healthy male who presented with acute fulminant hepatitis secondary to systemic Echovirus 25 (E25) infection, with concomitant Epstein–Barr virus (EBV) co-infection of recent onset. The diagnosis was established through multiplex PCR on cerebrospinal fluid, blood, stool, and nasopharyngeal aspirate, with serotype confirmation by the Italian National Institute of Health. The patient required intensive supportive care including therapeutic plasma exchange (TPE), continuous kidney replacement therapy (CKRT), and intravenous immunoglobulins (IGIV). Despite initial clinical deterioration and placement on the liver transplant list, the patient achieved complete hepatic recovery and was discharged after fourteen days of hospitalization without requiring transplantation. This case highlights the importance of prompt virological workup including enterovirus PCR in children presenting with acute liver failure of undetermined etiology and supports the use of extracorporeal therapies as a bridge to recovery.

## 1. Introduction

Pediatric acute liver failure (PALF) is a rare, rapidly progressive clinical syndrome that occurs in previously healthy children of all ages. The condition often results in rapid clinical deterioration and can be life-threatening without prompt intervention [1].

The incidence of PALF is not well defined; however, data from the United Network for Organ Sharing (UNOS) and the Organ Procurement and Transplantation Network (OPTN) indicate that acute liver failure accounts for approximately 11% of pediatric liver transplants. Notably, in about 30% of PALF cases, the underlying cause remains unidentified, with this percentage exceeding 60% among children aged 1–5 years. Children with liver failure of undetermined etiology have lower spontaneous survival rates and higher rates of transplantation and death compared to other diagnostic groups [1].

Viral infections are a significant cause of PALF. Common viral etiologies include hepatitis A, B, and E viruses, as well as herpes simplex virus (HSV) and Epstein–Barr virus (EBV) [2].

Human Enteroviruses (EVs) belong to the genus Enterovirus of the family Picornaviridae. They are small, non-enveloped, positive-sense single-stranded RNA viruses comprising more than 100 identified serotypes, classified into four species (A-B-C-D), which include polioviruses and non-polio enteroviruses (coxsackievirus A, coxsackievirus B, echovirus and enteroviruses) [3].

EVs are ubiquitous pathogens and a leading cause of self-limiting febrile, gastrointestinal or respiratory illnesses. Some EV genotypes are also responsible for serious diseases; in particular, in infants and young children, EVs can cause meningoencephalitis, myelitis, paralysis, myocarditis, sepsis-like syndrome, respiratory disease and acute liver failure [3]. Fulminant hepatitis caused by enteroviruses is rare but carries a high mortality rate, particularly in neonates and immunocompromised patients [4].

Given the rapid progression and high mortality associated with PALF, early recognition, intensive clinical care, and a multidisciplinary approach are crucial for improving clinical outcomes in affected children.

## 2. Case Report

We report the case of a nearly 4-year-old male who presented with acute hepatitis secondary to systemic Enterovirus infection and concurrent EBV co-infection.

His past medical history was unremarkable, with no recent travel, contact with wild animals, insect bites or unpasteurized milk consumption reported, and he was fully vaccinated according to the national vaccination program.

The child presented with sudden-onset high fever without other associated symptoms. Two days later, due to persistent fever, he was brought to the emergency department (ED). Blood tests revealed an elevated white blood cell count (WBC) of 24,000/mcl (63% lymphocytes), anemia (hemoglobin 8.8 g/dL, reference range 10.5–15.5 g/dL) mildly elevated C-reactive protein (CRP, 2.9 mg/dL, reference range < 0.5 mg/dL) and mild hypertransaminasemia (alanine aminotransferase (ALT) 140 UI/I nv < 33 UI/I, aspartate aminotransferase (AST) 193 UI/L); see Table 1. Initial infectious disease screening was suggestive of acute EBV infection, based on a qualitative serological assay positive for EBV IgM and negative for IgG; the patient was therefore discharged with a presumptive diagnosis of infectious mononucleosis.

Two days later, due to clinical deterioration and new-onset jaundice, he was brought again to the ED. Blood tests revealed markedly elevated transaminase levels (ALT 7026 U/L), associated with hyperbilirubinemia (total bilirubin 4.31 mg/dL; direct bilirubin 3.9 mg/dL) and coagulopathy (INR 2.65); see Table 1. The abdominal ultrasound showed hepato-splenomegaly, with hyperechoic aspect and without evidence of gross focal lesions, perihepatic and perisplenic free fluid. He was then admitted to the pediatric ward where vitamin K, broad spectrum antibiotic (piperacillin/tazobactam iv 100 mg/kg q8h) and acyclovir were started. His clinical status rapidly deteriorated; the patient became lethargic with evidence of progressive hepatic dysfunction (INR of 4.96) and hyperammonemia (131.6 µg/dL, reference range 27.2–102.0 µg/dL) requiring plasma and red blood cell transfusions. As his clinical course progressed toward acute fulminant hepatitis with neurological impairment, and liver transplantation considered as a potential therapeutic option, he was transferred to our tertiary-level hospital’s ICU.

At admission to our ED, tests revealed markedly elevated transaminase levels (ALT 3923 UI/L and AST 12,450 UI/L); see Table 1. The electroencephalogram (EEG) showed theta–delta activity with high-amplitude delta waves over the bilateral posterior regions; regional differentiation was only minimally appreciable, indicating globally delayed cerebral activity. The brain computed tomography (CT) scan was negative.

As required by the national protocol for the management of children with acute hepatitis of unknown etiology, hepatotropic viruses were investigated: hepatitis A virus (HAV), hepatitis C virus (HCV), hepatitis B virus (HBV), cytomegalovirus (CMV), herpes simplex virus (HSV) 1–2 and Parvovirus B19 serological analysis resulted negative for acute infection; EBV antibody profile was characterized by the presence of VCA-IgG, IgM and EBNA-IgG, and quantitative polymerase chain reaction (PCR) on peripheral blood detected an EBV viral load of 182,980 copies/mL; HIV serology was negative (Table 2). Bacterial infections were repeatedly ruled out with serial blood cultures. Hemophagocytic Lymphohistiocytosis (HLH)-related liver failure was excluded based on a ferritin value that dropped in 24 h from 1000 ng/mL to 500 ng/mL.

To rule out any other central nervous system infection, a lumbar puncture was performed: cerebrospinal fluid was clear and colorless with normal protein and glucose values; multiplex molecular assay returned positive for Enterovirus (viral load 4240 copies/mL) and negative for other viruses tested, including EBV; bacterial culture was also negative. Enterovirus was subsequently detected in stools, blood (30,476 cp/mL) and nasopharyngeal aspirate (149,862 cp/mL); see Table 2. In accordance with national guidelines, samples positive for Enterovirus were sent to the Italian National Institute of Health for serotyping, which identified the causative agent as Echovirus 25 (E25). Lymphocyte subset analysis, including B-cell, T-cell and natural killer (NK) cell counts and function, revealed no abnormalities, thereby excluding an underlying immunodeficiency.

According to our hospital’s acute liver failure (ALF) protocol, broad-spectrum antibiotic (piperacillin/tazobactam), pre-emptive antiviral therapy (acyclovir), a 10% dextrose infusion to manage hepatic hypoglycemia, N-acetylcysteine as an antioxidant, L-bioarginine to mitigate hyperammonemia and Fresh Frozen Plasma (FFP) along with a four-factor prothrombin complex concentrate to address coagulopathy were started.

Considering the worsening of EEG and rising ammonia levels, the child was started on therapeutic plasma exchange (TPE), a modality employed in ALF to remove circulating cytokines, protein-bound toxins, and autoantibodies, thereby providing the native liver with the opportunity to recover. Simultaneously, continuous kidney replacement therapy (CKRT) was commenced using Continuous Veno-Venous Hemofiltration (CVVH), a modality in which blood is continuously passed through a hemofilter and solutes are removed via convection-driven ultrafiltration. Intravenous immunoglobulins (IGIV) were administrated at 400 mg/kg/day for three consecutive days intravenously (IV).

Clinical status deteriorated over the first 24 h, with the patient remaining persistently lethargic and exhibiting signs of neurological irritability. Consequently, the decision was made to place the patient on the liver transplant list, with a Pediatric End-Stage Liver Disease (PELD) score of 26.

After two sessions of TPE and four days of CKRT, the patient’s clinical status progressively improved; he became alert and responsive, with restoration of hepatic function, and was consequently removed from the transplant list.

During hospitalization, serial blood tests and abdominal ultrasound examinations demonstrated complete normalization of laboratory and imaging findings; he was discharged after fourteen days of hospitalization. The patient attended a follow-up visit three weeks after discharge: the child had returned to his normal daily activities, and blood tests including liver transaminases, bilirubin levels, WBC count, and CRP levels had normal values.

## 3. Discussion

This case is of particular interest given the rare association of Echovirus 25 (E25) with fulminant hepatitis in childhood. To our knowledge, only one previous pediatric case of acute liver failure caused by E25 has been reported in the literature, in a 2-year-old previously healthy child, in whom serological cross-reactivity with hepatitis A virus (HAV) initially led to misdiagnosis—underscoring both the diagnostic challenge and the likely underestimation of E25-related hepatic disease [5]. In infants and children, E25 has previously been reported in association with encephalitis and aseptic meningitis [6] and has rarely been identified as a cause of acute liver failure in the pediatric population.

Between 2022 and 2023, an increase in severe cases caused by a new highly pathogenic E11 variant (new lineage 1) was reported in Europe, prompting a World Health Organization alert [5]. The original report from France described nine cases of severe neonatal sepsis with liver failure between July 2022 and April 2023, of which seven were fatal—all associated with a new recombinant variant of E11 [6]. This outbreak, rapidly detected across multiple European countries including Italy, France, Germany, Spain, Sweden, and the United Kingdom, led to a formal alert in the EU Early Warning and Response System and subsequent inclusion in the ECDC Communicable Disease Threat Report, highlighting the potential of specific enterovirus variants to cause life-threatening neonatal disease [6]. The Italian case of fulminant E11 hepatitis in non-identical dichorionic twin brothers, reported in April 2023 and genetically closely related to the French outbreak strains, further confirmed the international spread of this new E11 lineage [7,8]. Molecular characterization through whole-genome sequencing revealed that the pathogenicity of this new variant is likely linked to recombination events involving both structural and non-structural genomic regions [9]. More recently, Ikuse et al. described a cluster of neonatal acute liver failure cases associated with E11 infections in Japan between August and November 2024, further confirming the global relevance of this emerging serotype [5].

A study conducted in children aged ≤12 years identified viral infections as the most common cause of infectious liver damage, EBV, CMV and EV being the main etiological agents [10]. A retrospective surveillance study conducted across the European Union highlighted a wide circulation of non-polio enteroviruses in Europe, mostly affecting young children and leading to neurological symptoms. Outbreaks due to EV-D68, EV-A71, coxsackievirus A6 (CVA6) and different echoviruses (i.e., E30) have been frequently reported [11].

The pathophysiology of Enterovirus-induced hepatic failure involves direct viral cytotoxicity, immune-mediated hepatocyte destruction, and inflammation driven by damage-associated molecular patterns (DAMPs) [12]. Hepatocyte necrosis occurs either through a direct cytopathic effect or as a consequence of immune-mediated injury. Certain viruses, including enteroviruses, can directly induce hepatocyte death through viral replication and cytotoxicity involves widespread viral replication in hepatocytes, leading to cellular necrosis and an intense inflammatory response. This process can rapidly progress to fulminant liver failure, particularly in hosts with underdeveloped or compromised immune systems [13].

The resulting hepatocyte death triggers the release of DAMPs, including high mobility group box 1 (HMGB1), histones, and mitochondrial DNA (mtDNA). These molecules activate pattern recognition receptors (PRRs) on Kupffer cells and dendritic cells, further amplifying the immune response. The subsequent release of pro-inflammatory cytokines such as tumor necrosis factor-alpha (TNF-α), interleukin-1β (IL-1β), and interleukin-6 (IL-6) creates a hyperinflammatory state that exacerbates liver injury and contributes to systemic complications [14,15].

Advancements in molecular diagnostics, including PCR-based assays, have facilitated the identification of Enterovirus in patients presenting with severe hepatic symptoms [16]. These tools are critical for differentiating viral hepatitis from other causes, such as autoimmune or drug-induced liver injury. A landmark study [17] demonstrated the ability of Enterovirus to induce hepatocellular necrosis in neonates. These findings underscored the importance of early virological detection and timely supportive intervention to mitigate severe outcomes. In our patient, the systematic application of multiplex molecular testing across multiple anatomical compartments—CSF, blood, stool and nasopharyngeal aspirate—enabled the identification of E25 as the causative agent. Similarly, a case series [18] highlighted coxsackievirus B as a predominant cause of fulminant hepatic failure in neonates, emphasizing the role of serotyping and molecular diagnostics for accurate pathogen identification.

Another critical aspect in the diagnostic workup of PALF is the exclusion of Hemophagocytic Lymphohistiocytosis (HLH), a hyperinflammatory syndrome often linked to genetic mutations affecting immune regulation. Mutations in genes such as PRF1, UNC13D, STX11, and STXBP2 have been implicated in familial forms of HLH, leading to dysregulated immune responses [19]. While HLH primarily manifests as a systemic hyperinflammatory condition, its impact on hepatic function is profound, often leading to cholestasis, hepatomegaly, and fulminant liver failure. The excessive activation of macrophages and T cells, coupled with the overproduction of cytokines such as IFN-γ, IL-6, and TNF-α, contributes to severe hepatic inflammation and necrosis [20]. Distinguishing HLH-related liver failure from other causes of PALF remains challenging due to overlapping clinical features. Biomarkers such as ferritin, soluble IL-2 receptor (sCD25), and CXCL9 have been proposed as potential indicators of HLH-related immune activation in hepatic dysfunction [21]. In our patient HLH-related liver failure was excluded based on the rapid drop in ferritin and normalization of white blood cell count. Given the potential genetic predisposition, early genetic screening for mutations in HLH-associated genes, combined with immune profiling, is recommended in pediatric patients presenting with acute liver failure of infectious origin. Early identification of these mutations can facilitate timely and targeted therapeutic strategies.

In our case, EBV co-infection was documented; however, the detection of EBNA-IgG at the time of ICU admission indicates that primary EBV infection had occurred approximately 3–4 weeks prior to the onset of fulminant hepatitis, as EBNA-IgG typically becomes detectable only several weeks after primary EBV infection. This serological profile, together with a quantitative EBV viral load of 182,980 cp/mL consistent with a resolving primary infection, led us to focus diagnostic and clinical attention primarily on Echovirus 25 as the causative agent. Nevertheless, a contributory role of EBV in the pathogenesis of hepatic injury cannot be entirely excluded. Indeed, evidence suggests that viral co-infections may amplify EBV-induced liver damage through synergistic immunopathological mechanisms, and the concurrent immune activation driven by two distinct viral pathogens may have contributed to the severity of the hepatic injury observed in this patient [20].

Despite advances in intensive care, treatment options for Enterovirus-induced fulminant hepatitis remain limited. IGIV, although utilized in certain cases, still requires further investigation to fully assess its efficacy and the effectiveness of other antiviral therapies. A review on therapeutics for fulminant hepatitis caused by enteroviruses in neonates discusses potential treatment strategies, emphasizing the need for more research in this area [20].

Supportive care remains the cornerstone of the treatment and ranges from TPE and CKRT to even liver transplantation in unresponsive and severe cases. TPE can eliminate inflammatory mediators, autoantibodies, protein-bound toxins, and other small plasma molecules, as well as correct coagulopathy.

CKRT effectively removes smaller, water-soluble molecules, such as ammonia, while also addressing acute kidney injury (AKI) and fluid overload, both of which are frequently observed in patients with ALF. It is increasingly used in PALF, as a bridge to recovery or liver transplantation, particularly in critically ill patients or when renal dysfunction is present. CKRT provides slow and continuous renal support which is better tolerated than intermittent hemodialysis, and helps manage fluid balance, correct electrolyte disturbances, remove toxins, and support metabolic functions [22,23]. A recent paper showed that CKRT with regional citrate anticoagulation (RCA), as applied in our case, was easily applied in pediatric patients with ALF without citrate toxicity or metabolic complications suggesting its potential adoption as part of standard care protocols in specialized pediatric centers [24]. Combining TPE and CKRT may offer a more effective approach to stabilizing patients with ALF compared to the use of either modality alone [25].

The prognosis of enterovirus-associated fulminant hepatitis is variable and depends on the patient’s age, immune status, viral serotype, and timeliness of supportive intervention. Mortality is highest in neonates, where rates exceeding 70% have been reported during the recent E11 outbreak in Europe [6]. In older children, spontaneous hepatic recovery, as observed in our patient, may occur with aggressive extracorporeal support, potentially avoiding the need for liver transplantation.

In our case, after two sessions of TPE and four days of CKRT, the patient achieved complete hepatic and neurological recovery, was removed from the transplant list, and was discharged after 14 days of hospitalization. Follow-up three weeks after discharge confirmed normalization of all laboratory parameters and full return to daily activities.

## 4. Conclusions

In conclusion, enterovirus-associated fulminant hepatitis is an uncommon but severe and potentially life-threatening condition that requires prompt recognition and timely intervention. The systematic inclusion of enterovirus PCR testing across multiple anatomical compartments—including CSF, blood, stool, and nasopharyngeal secretions—in the routine diagnostic workup of PALF of undetermined etiology enables prompt pathogen identification and facilitates the timely initiation of targeted supportive therapy.

This case also highlights the diagnostic complexity of viral co-infections in the pediatric setting, where careful interpretation of serological profiles—including the timing of antibody seroconversion—is essential to correctly attribute the primary causative agent. Furthermore, it underscores the potential role of extracorporeal therapies, including TPE and CKRT, as an effective bridge to hepatic recovery in children with severe ALF, potentially avoiding the need for liver transplantation.

## Figures and Tables

**Table 1:** Timeline laboratory parameters during hospitalization.

Timepoint	ALT (U/L) [nv < 33]	AST (U/L) [nv < 40]	INR [0.8–1.2]	Total Bilirubin/Direct Bili (mg/dL)	CRP (mg/dL) [<0.5]	WBC (/mcL)
Day 1 (1st ED visit)	140	193	Unknown	N/A	2.9	24,000
Day 3 (2nd ED visit)	7026	Unknown	2.65	4.31/3.9	Unknown	Unknown
Day 5 (ICU admission)	3923	12,450	3.92	5.92/4.5	1.82	31,500
Day 6 (ICU)	2319	5755	3.22	7.44/5.12	0.84	35,200
Day 7 (ICU)	1054	2389	1.65	6.45/4.64	0.80	27,220
Day 10 (ward)	517	484	1.03	3.28/2.45	0.47	4590
Day 14 (discharge)	64	62	0.99	0.89/0.1	0.06	5910

ALT: alanine aminotransferase; AST: aspartate aminotransferase; INR: international normalized ratio; Bili: bilirubin; CRP: C-reactive protein; WBC: white blood cell count; N/A: not available at that timepoint; ED: emergency department.

**Table 2:** Virological data: serology and PCR results.

Pathogen/Test	1st ED Visit (Day 1)	ICU Admission (Day 5)
EBV VCA-IgM	Positive (qualitative)	Positive
EBV VCA-IgG	Not performed	Positive
EBV EBNA-IgG	Not performed	Positive
EBV PCR (blood)	Not performed	182,980 cp/mL
Enterovirus PCR (CSF)	Not performed	Positive (4240 cp/mL)
Enterovirus PCR (blood)	Not performed	30,476 cp/mL
Enterovirus PCR (NPA)	Not performed	149,862 cp/mL
Enterovirus PCR (stool)	Not performed	Positive
HAV, HBV, HCV IgM-IgG	Not performed	Negative
CMV, HSV 1–2 IgM-IgG	Not performed	Negative
Parvovirus B19 IgM-IgG	Not performed	Negative
HIV IgG	Not performed	Negative

EBV: Epstein–Barr virus; VCA: viral capsid antigen; EBNA: Epstein–Barr nuclear antigen; CSF: cerebrospinal fluid; NPA: nasopharyngeal aspirate; HAV/HBV/HCV: hepatitis A/B/C virus; CMV: cytomegalovirus; HSV: herpes simplex virus.

## Data Availability

No new data were created or analyzed in this study.

## Abbreviations

The following abbreviations are used in this manuscript:

PALF	Pediatric acute liver failure
EBV	Epstein–Barr virus
EVs	Human Enteroviruses
HSV	Herpes simplex virus
ED	Emergency department
CRP	C-reactive protein
ALT	Alanine aminotransferase
AST	Aspartate aminotransferase
EEG	Electroencephalogram
FFP	Fresh Frozen Plasma
PCR	Polimerase chain reaction
TPE	Therapeutic plasma exchange
CKRT	Continuous kidney replacement therapy
E25	Echovirus 25
IG	Human immunoglobulin
IV	Intravenously
HLH	Hemophagocytic Lymphohistiocytosis
